# Characterization of Seasonal Trivalent Influenza Vaccine Formulated with Different Ratios (*v*/*v*) of Squalene-Containing IB160 Adjuvant in One- or Two-Vial Presentations

**DOI:** 10.3390/vaccines14090789

**Published:** 2026-09-09

**Authors:** Daniela Cajado-Carvalho, Paulo Newton Tonolli, Verônica de Moraes Manzato, Lívia Mendonça Munhoz Dati, Mahyumi Fujimori, Patrícia Antonia Estima Abreu, Christian Savio Silva, Vitor Anselmo Sakihara, Bianca Pereira Carvalho Holanda, Douglas Gonçalves de Macedo, Priscila Comone, Alexandre Bimbo, Esper George Kallás, Ricardo das Neves Oliveira, Milena Apetito Akamatsu, Paulo Lee Ho

**Affiliations:** Bioindustrial Center, Butantan Institute, and Butantan Foundation, Sao Paulo 05503-900, SP, Brazil; daniela.carvalho@fundacaobutantan.org.br (D.C.-C.); paulo.tonolli@fundacaobutantan.org.br (P.N.T.); veronica.manzato@fundacaobutantan.org.br (V.d.M.M.); livia.dati@fundacaobutantan.org.br (L.M.M.D.); mahyumi.fujimori@fundacaobutantan.org.br (M.F.); patricia.aniz@butantan.gov.br (P.A.E.A.); christian.silva@fundacaobutantan.org.br (C.S.S.); vitor.sakihara@fundacaobutantan.org.br (V.A.S.); bianca.carvalho@fundacaobutantan.org.br (B.P.C.H.); douglas.macedo@fundacaobutantan.org.br (D.G.d.M.); priscila.comone@fundacaobutantan.org.br (P.C.); alexandre.bimbo@fundacaobutantan.org.br (A.B.); esper.kallas@butantan.gov.br (E.G.K.); ricardo.oliveira@fundacaobutantan.org.br (R.d.N.O.)

**Keywords:** TIV, adjuvant IB160, formulation strategies

## Abstract

Background/Objectives: Adjuvanted influenza vaccines are critical to enhance immune responses in high-risk populations, such as the elderly. While initial studies relied on a two-vial system with antigen and adjuvant mixing prior to immunization, this study evaluated the feasibility, immunogenicity, and 12-month stability of a one-vial formulation combining a Trivalent Influenza Vaccine (TIV) with the locally produced squalene-based oil-in-water emulsion IB160. Methods: Formulations were evaluated by testing TIV:IB160 (*v*/*v*) ratios (1:1, 2:1, 3:1) in an animal model, measuring HI antibodies as the primary immunogenicity metric, complemented by qualitative strain-specific total IgG screening by ELISA. One-vial and two-vial strategies were also compared regarding immune responses. Formulations stabilities were evaluated at 6.0 °C ± 2.0 °C in one-and two-vial systems by monitoring physicochemical features and their antigenic responses, measuring hemagglutinin content of the formulated vaccines, and long-term HI antibody responses. Results: The 2:1 and 1:1 (*v*/*v*) formulation ratios (15 µg TIV:IB160) induced significantly higher HI titers across all tested strains (A/H3N2/Thailand, A/H1N1/Victoria, and B/Austria) when compared to non-adjuvanted TIV or adjuvanted TIV in the 3:1 ratio. Both the one and two-vial strategies showed significantly higher HI antibody responses than the non-adjuvanted control group (*p* < 0.05), with no significant differences observed between the delivery formats. Indeed, the one-vial formulation with 1:1 and 2:1 ratios remained stable for 12 months, preserving TIV antigenicity, IB160 emulsion integrity, and robust immunogenicity. Conclusions: One- and two-vial formulations are viable, stable, and highly immunogenic alternatives for adjuvanted TIV in 2:1 and 1:1 (*v*/*v*) formulation ratios (15 µg TIV:IB160). One-vial format simplifies immunization logistics, minimizes operational errors, and is more indicated for routine use, while the two-vial system remains a versatile asset for emergency responses.

## 1. Introduction

Influenza is an important contagious respiratory disease with a high global incidence, causing approximately 290,000 to 650,000 deaths annually, in addition to morbidity, according to the World Health Organization (WHO) [1,2]. The disease is primarily caused by Influenza A and B viruses, which are responsible for severe clinical outcomes and seasonal epidemics [1,3,4]. Due to the potential severity of this disease, the WHO recommends global immunization programs, prioritizing populations over 60 years old to reduce severe cases. Nevertheless, this group also presents a reduced immune response to influenza vaccines [5]. To overcome this limitation, adjuvants can be incorporated into vaccine formulations to stimulate both the innate and adaptive immune systems [6,7]. Adjuvants not only enhance overall efficacy but can also provide a crucial dose-sparing effect, reducing the amount of antigen required per dose or the number of immunizations needed to achieve protective titers [6,7]. Although aluminum salts have been the traditional adjuvants of choice for decades, squalene-based oil-in-water emulsions, such as MF59, AS03, and AF03, have emerged as safe and highly effective alternatives, specifically proven to boost immunogenicity in both seasonal and pandemic influenza vaccines [8,9].

Recognizing the importance of these advanced adjuvant emulsions for public health, especially for pandemic influenza preparedness, Instituto Butantan initiated a strategic development partnership with the Infectious Disease Research Institute (IDRI), with support from BARDA and WHO, to produce its own squalene-based oil-in-water emulsion adjuvant, designated IB160 [10]. Emulsions of this type, such as MF59, are known to stimulate a robust, mixed Th1/Th2 humoral response through local macrophage recruitment and DAMPs (Damage-Associated Molecular Patterns) [11,12,13]. This partnership enabled Instituto Butantan to optimize manufacturing parameters and validate the local production of IB160, to be combined with specific antigens for the development of adjuvanted vaccines.

The strategic value of this adjuvant was first demonstrated in the development of a vaccine against the poorly immunogenic H7N9 pandemic avian influenza virus [14]. Pre-clinical studies revealed that combining the H7N9 strain with the IB160 adjuvant significantly enhanced the immune response and reduced the antigen dose required to induce protective neutralizing antibodies in animal models after two doses [10,14]. Furthermore, the IB160 adjuvant proved highly stable, maintaining its physicochemical characteristics for up to 5 years under refrigerated conditions (5.0 °C ± 3.0 °C) [10]. These pre-clinical results led to a Phase I randomized, double-blind clinical trial, which confirmed the dose-sparing effect and robust immunogenicity of the H7N9+IB160 formulation after two doses [15].

Building upon this validated platform, Instituto Butantan is currently advancing its pandemic preparedness by developing an adjuvanted vaccine against H5Nx clade 2.3.4.4b avian influenza strains. Using the established egg-based platform and the established pilot production of the IB160 adjuvant, a vaccine candidate showed promising results, although two doses were required to achieve appropriate HI titers in sera from immunized animals [16].

Beyond pandemic preparedness, the IB160 adjuvant can also be combined with a seasonal Trivalent Influenza Vaccine (TIV) with the aim of enhancing the immune response in the elderly population, providing greater protection for this high-risk group of people as an alternative to high-dose TIV formulations, such as Efluelda, which contains 60 μg of antigen per dose instead of the 15 μg of antigens per dose used in conventional TIV vaccines.

This study evaluates the feasibility of a one-vial (ready-to-use) formulation, which is more appropriate for seasonal TIV combined with the IB160 adjuvant. We investigated different antigen-to-adjuvant ratios (1:1, 2:1, and 3:1 *v*/*v*) and evaluated both standard (15 µg) and higher (30 µg) antigenic doses per strain as possible alternative formulations. Furthermore, immunogenicity was primarily evaluated through functional hemagglutination inhibition (HI) antibody titers, complemented by qualitative total IgG antibody screening, in addition to a 12-month formulation stability assessment.

## 2. Materials and Methods

### 2.1. Study Formulations

#### 2.1.1. Production of Adjuvant IB160

The oil-in-water emulsion IB160 was prepared as previously described [10] under Good Manufacturing Practice (cGMP) conditions. Briefly, for the adjuvant production, the aqueous phase (citrate buffer and polysorbate 80) and the oil phase (squalene oil and sorbitan trioleate) were mixed together at high speed to form a pre-emulsion. This pre-emulsion solution was then emulsified in a high-pressure homogenizer until reaching the desired particle size, monitored by dynamic light scattering assay. The emulsion was filtered through a 0.22 μm PVDF filter and 1.5 mL aliquots of the emulsion were filled into 7.5 mL glass vials and sealed with polytetrafluoroethylene (PTFE)-coated chlorobutyl stoppers and aluminum caps. Vials containing the adjuvant were stored at 2–8 °C until use.

#### 2.1.2. Production of Influenza Antigens

The monovalents from seasonal Influenza strains (split and inactivated) used in this study were produced at an industrial scale under cGMP. The composition of seasonal 2024’s TIVs evaluated followed the WHO composition recommendation for the 2024 Southern Hemisphere campaign: A/Thailand/8/2022, IVR-237 (H3N2) (batch 2400172/00), A/Victoria/4897/2022, IVR-238 (H1N1) (batch 2301920/00), and B/Austria/1359417/2021, BVR-26 (batch 2302646/00). The antigens were stored at 2–8 °C until use.

#### 2.1.3. Formulations

Formulations were prepared according to Table 1. For the adjuvanted formulations, two distinct strategies were explored: filling the TIV and IB160 adjuvant into two separate vials (2-vial format) or combining them in one vial (1-vial format). Additionally, TIV:IB160 volume ratios were also tested (1:1 and 3:1 in a 0.5 mL volume/dose, using 15 µg/dose; and 2:1 in a 0.75 mL volume/dose, using 15 µg or 30 µg/dose). The control formulations were also prepared (PBS pH 7.2, IB160, 15 µg TIV and 30 µg TIV).

### 2.2. Vaccine Immunization

The experimental protocols were approved by the Committee on Ethics in Animal Experimentation of Butantan Institute (CEUAIB), protocol number 8580020424. The study was performed according to the guidelines outlined by the Brazilian National Council for Control of Animal Experimentation (CONCEA). Male rats (Wistar strain, weighing 300–350 g) aged three months were purchased from ANILAB Veterinary Laboratory (Paulínea, SP, Brazil). Male Wistar rats were selected as the animal model for two primary reasons: first, to obtain sufficient biological sample volumes required for multi-parameter immunological evaluations without increasing animal numbers; second, to maintain consistency and direct comparability with our historical datasets evaluating IB160-adjuvanted influenza candidate vaccines [10,16]; third, we immunized the animals with the same route and volume used in human immunizations, that is 0.5 mL and via intramuscular (IM). In this case, the volume was divided in two, and one volume was applied in one muscle in the hind legs via IM while the other volume was applied in the muscle of the other hind leg.

Groups of six animals, housed in two cages containing three animals each, were im-munized once (no booster) with TIV doses of 15 or 30 µg in the presence or absence of the IB160 adjuvant using 1:1, 2:1 or 3:1 TIV:IB160 (*v*/*v*) proportions, resulting in 12 experimental groups with 72 animals per assay periods (0, 3, 9 and 12 months) (Table 1) as schematically illustrated in Figure S1. A group size of six animals was selected to provide sufficient serum volume for the HI assay while allowing statistical comparison of immune responses among groups using one-way ANOVA followed by Tukey’s multiple comparison test. In accordance with the 3Rs principles (Reduction), shared negative control groups (PBS and IB160 alone) were used across comparative analyses to minimize redundant animal testing. Formulations were prepared in 1-vial or 2-vial formats; for the 2-vial formulation, mixing was performed immediately prior to immunization. A vaccine formulation volume of 0.5 mL (1:1 and 3:1 formulations) or 0.75 mL (2:1 formulation) (Table 1), was administered intramuscularly into the quadriceps muscle of the animal’s hind limb. Each dose was divided into two inoculations in the right and left hind limbs (0.25 or 0.375 mL/muscle, respectively, for the 0.5 mL or 0.75 mL formulation volumes). Twenty-one days after immunization, the animals were anesthetized with 90 mg/kg of ketamine and 10 mg/kg of xylazine. Exsanguination was performed via cardiac puncture, and the blood was collected and incubated at 37 °C for 30 min after a conditioned procedure at 4 °C for 1 h for serum collection. Samples were centrifuged at 5000 rpm, 4 °C for 20 min, and the sera were collected, aliquoted, and stored at −20 °C for subsequent analysis.

### 2.3. Immunological Evaluation—Hemagglutination Inhibition (HI) Assay

Biological variability and protective functional antibody titers were individually assessed using the HI assay (*n* = 6 animals/group). The sera from immunized animals were treated with neuraminidase from *Vibrio cholerae* (Sigma-Aldrich, St. Louis, MO, USA) at 37 °C for 18–20 h, followed by heat inactivation at 56 °C and adjustment to a final 1:5 dilution in saline solution to remove nonspecific hemagglutination inhibitors. Prior to the assay, the viral suspension was titrated to standardize the working dose. One hemagglutination unit (1 HAIV) was defined as the highest dilution inducing complete hemagglutination, which served as the baseline to prepare an 8 HAIV/25 µL suspension, to yield a final concentration of 4 HAIV/50 μL of antiserum inhibition volume for each well. The pretreated sera were two-fold serially diluted in the assay plates, starting at an initial dilution of 1:10. The dilutions were then mixed with the standardized viral suspension for 4HAIV/50 μL and incubated for 15 min at room temperature. Subsequently, 50 μL of a 1% guinea-pig erythrocyte suspension was added to the wells. The HI antibody titer was determined as the highest serum dilution capable of inhibiting 90–100% of hemagglutination after a 1 h incubation. For data analysis, the arithmetic mean of the individual animal values within each group was used; serum samples with no detectable HI activity were arbitrarily assigned a titer of 5. HI titers > 40 were considered seroprotective, a threshold conventionally associated with a 50% protective efficacy in the vaccinated population [17]. Log_2_ TIV-specific hemagglutination inhibition titers were analyzed by one-way ANOVA followed by Tukey’s multiple comparison test using GraphPad Prism Software, version 5.03 for Windows. Significant differences were indicated (* *p* ≤ 0.05, ** *p* ≤ 0.01, *** *p* ≤ 0.001, **** *p* ≤ 0.0001).

### 2.4. Evaluation of Formulation by Physicochemical Analysis

The vials containing the formulations were stored at 6.0 °C ± 2.0 °C for a stability study up to 12 months. The samples were analyzed just after the filling process (initial point of stability) and after 3, 9, and 12 months for the long-term stability study. The samples were evaluated for general appearance, pH, squalene content, particle size, and concentration when formulated with IB160. TIV hemagglutinin content for each Influenza strain was also determined, as described below.

It is important to note that the IB160 bulk control sample used to evaluate physicochemical stability corresponds to the stock adjuvant emulsion stored without PBS or any other addition, as it represents the concentrated adjuvant component prior to combination with the antigen. Therefore, to directly compare the particle concentration (NTA) and squalene content (HPLC) of the adjuvant component across different ratios against the undiluted IB160 bulk control, the measured values for the 1:1 and 2:1 formulations were adjusted using their respective dilution factors (1:2 for the 1:1 *v*/*v* ratio and 1:3 for the 2:1 *v*/*v* ratio).

#### 2.4.1. IB160 Adjuvant: Appearance and Particle Size

The appearance of the IB160 emulsion used for the stability study was evaluated, considering the homogeneity and general aspects. The size measurement of the IB160 particles was performed by dynamic light scattering (DLS) analysis, using a Malvern Zetasizer NanoS (Malvern Panalytical, Malvern, UK), diluting the IB160 emulsion in filtered ultrapure water. The intensity of scattering values to obtain Z-average (particle diameter, d.nm) was obtained after three measurements of 60 s each, setting the sample as an emulsion, diluted in water and pre-incubating the sample for ten seconds at 25 °C. The acceptance criteria for quality were set at the lower and upper limits of acceptable size, 130 and 190 d.nm, respectively. Besides, the polydispersity index was also analyzed by DLS, considering PdI < 0.2 as a monodisperse solution.

#### 2.4.2. IB160 Adjuvant: Particle Size and Concentration Analysis by NTA

The Nanoparticle Tracking Analysis (NTA) was performed using a NanoSight NS300 (Malvern Panalytical, Malvern, UK) equipped with a 532 nm green laser and a syringe pump for controlled sample injection. The instrument operates within a detection range of 10 to 2000 nm and concentrations of 10^6^ to 10^9^ particles/mL. For each sample, five videos (60 s/video) were captured to record individual particle movement, allowing the software to determine both size distribution and concentration.

#### 2.4.3. IB160 Adjuvant: Determination of Squalene Content by HPLC

Squalene content in IB160 emulsion was determined by high-performance liquid chromatography (HPLC) analyses. Analyses were performed using an Agilent 1260 Infinity II with diode array detection (HPLC-DAD) equipped with a G7129C autosampler and a G7117C DAD HS detector (Agilent, Santa Clara, CA, USA), and InfinityLab Poroshell 120EC-C18 (3.0 × 100 mm, 2.7 μm particle diameter, maximum pressure of 1000 bar) column. Parameters used were: acetonitrile (HPLC grade) isocratic elution at 100% at 1.0 mL/min flow rate, with the chromatography column operating at 30 °C, 10 µL injection volume, DAD set to 208 nm detection, and a 10 min total run time. For the calibration curve, the squalene reference standard solution was prepared at 1 mg/mL in a 50 mL volumetric flask, using 2-propanol (HPLC grade) as diluent. After that, five calibration point dilutions were prepared in the 100–500 µg/mL range. Analyte quantification was carried out using the calibration curve. The analyte prepared in 2-propanol was analyzed in triplicate. After mixing, samples were filtered through 0.45 µm Polyvinylidene fluoride (PVDF) membrane filters and transferred to HPLC vials. IB160 in formulations were analyzed the same way, using Sigma Reference Standard (cat: W002467). OpenLab CDS v2.7 was the software used for system control and data analysis. For statistical evaluation, GraphPad Prism Software, version 5.03 for Windows was used to determine squalene content variability at different formulations, stability conditions, and incubation times.

#### 2.4.4. TIV: Determination of HA Content

Single Radial Immunodiffusion (SRID) technique was performed to determine the hemagglutinin (HA) content following the protocol of Schild and colleagues [18]. The average concentration of HA, expressed as µg/mL, was calculated. Gels were prepared with 1% (*w*/*v*) Agarose SeaKem HE containing strain-specific antisera (NIBSC 23/100 for A/Victoria, TGA AS450 for A/Thailand, and NIBSC 21/326 for B/Austria). Reference antigens (NIBSC 22/320, TGA 2023/145B, and NIBSC 21/316, respectively) were prepared based on the nominal vaccine concentrations. Test samples and reference standards were treated with Zwittergent 3–14 detergent and prepared in triplicate using specific dilution series (1/1, 3/4, 1/2, and 1/4 for reference; 1/1, 3/4, 1/2, and 1/3 for samples). The solutions were randomly loaded into a 49-well grid punched in the agarose plates, allowing the analysis of up to three samples per plate. Following incubation in a humidified chamber (18–22 h), the plates were washed with PBS and purified water, dried in an oven, and stained with Coomassie Brilliant Blue G-250. Precipitation halo diameters were captured using a Symbiosis scanner Epson Expression 13000XL (Seiko Epson Corporation, Suwa, Japan), measured via Protocol3 software, and statistically analyzed using Combistats software version 1.2.2 to calculate the HA content by the slope-ratio method.

#### 2.4.5. Evaluation of pH

For pH determination, formulated vials were equilibrated to room temperature. A SevenExcellence pH meter (Mettler Toledo, Columbus, OH, USA) was calibrated using Certipur^®^ buffer solutions prior to analysis. Each sample was homogenized and measured using a pH electrode. Samples were analyzed in duplicate, and the arithmetic mean was recorded.

## 3. Results

### 3.1. Evaluation of TIV:IB160 Ratios in Two-Vial Formulations

The safety and efficacy of the adjuvant IB160 was previously demonstrated in 0.5 mL adjuvanted pandemic influenza vaccines using two-vial formulations at a 1:1 (*v*/*v*) ratio [10,15]. For this reason, we first evaluated the seasonal TIV from the 2024 Brazilian Influenza Campaign adjuvanted with IB160 emulsion using two-vial presentations. We tested three volume ratios (1:1, 2:1, and 3:1, TIV:IB160, Table 1) and statistically compared their performances to TIV alone and also with each other based on HI titers. Moreover, we tested higher TIV doses (30 µg) to possibly boost the immune response, considering that poor responders might need higher doses of antigen to obtain an effective immunization. Regarding the study design, all experimental groups were evaluated concurrently within the same study under identical conditions, with data presented in distinct subplots for analytical clarity. In accordance with the 3Rs principles (Reduction), shared control groups were utilized across these visual comparisons to avoid redundant animal testing. To accommodate dosage volumes (0.50 mL vs. 0.75 mL) without causing local muscle strain, immunizations were administered bilaterally across both hind limbs (0.25 mL or 0.375 mL/site), which remained strictly within recommended veterinary limits for adult Wistar rats.

**Figure 1:**
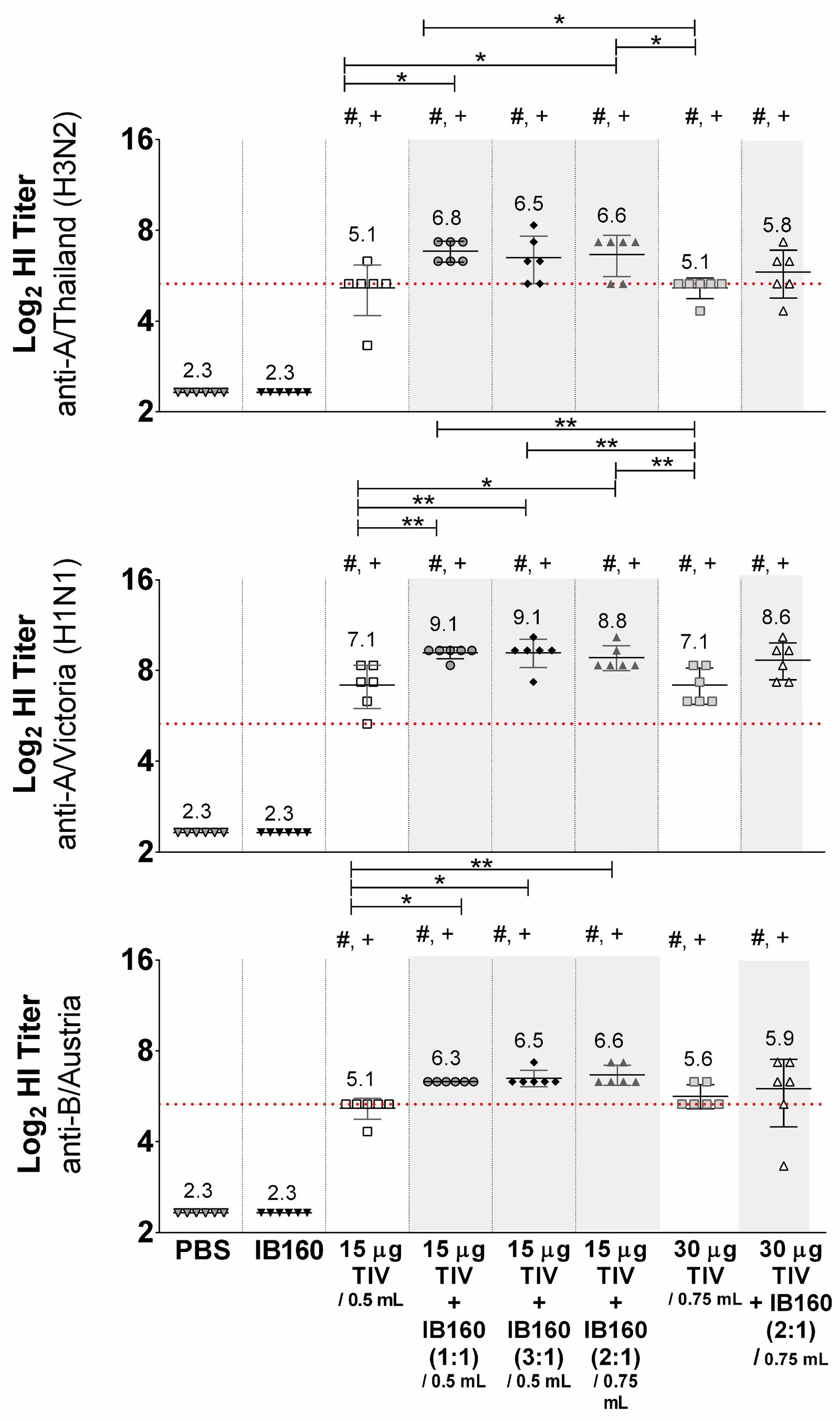
Two-vial formulations: Effects of different TIV:IB160 ratios (*v*/*v*) on HI titers. The sera from immunized animals were collected 21 days after immunization. Each symbol represents one animal per group. The horizontal red dotted line represents the HI titer threshold; equal to or higher than log_2_ 5.22 (corresponding to 1:40) is associated with seroprotection. Comparing the vaccination effect between various formulations, an asterisk * indicates a significant difference by one-way ANOVA (* *p* ≤ 0.05, ** *p* ≤ 0.01). # and + indicate statistical significance compared to PBS and IB160, respectively. Numbers above the groups represent GMT values.

Initial qualitative screening of total IgG binding antibodies (Figure S2) indicated that the 1:1 and 2:1 formulations (15 µg TIV + IB160) produced a more pronounced increase in IgG titers across all tested influenza strains compared to the non-adjuvanted group, whereas the response induced by the 3:1 formulation was strain-dependent. To rigorously evaluate the functional humoral immune response, we performed Hemagglutination Inhibition (HI) assays as the primary indicator of immunogenicity (Figure 1). A statistically significant increase in HI titers (log_2_) across all influenza strains was exclusively observed in animals immunized with the 1:1 and 2:1 formulations (15 µg TIV + IB160) compared to TIV alone, confirming the robust adjuvant effect of the IB160 emulsion at these ratios. Also, for formulations with a 3:1 ratio (using a 15 µg TIV dose), the statistical significance was again strain-dependent, being observed only for the H1N1 and B strains (*p*< 0.05), but not for the A/Thailand strain. Increasing the antigen dose to 30 µg without adjuvant yielded no significant benefits over the 15 µg non-adjuvanted dose in HI titers. Furthermore, combining the higher 30 µg TIV dose with IB160 did not result in a significant increase in HI titers when compared to 30 µg TIV alone. Considering these findings, the 1:1 and 2:1 (15 µg TIV + IB160) formulations were selected to proceed for further analysis.

### 3.2. Two-Vial or One-Vial Formulations Are Viable Alternatives for TIV Adjuvanted Vaccine and Show Superior Results When Compared to Non-Adjuvanted TIV Vaccine

**Figure 2:**
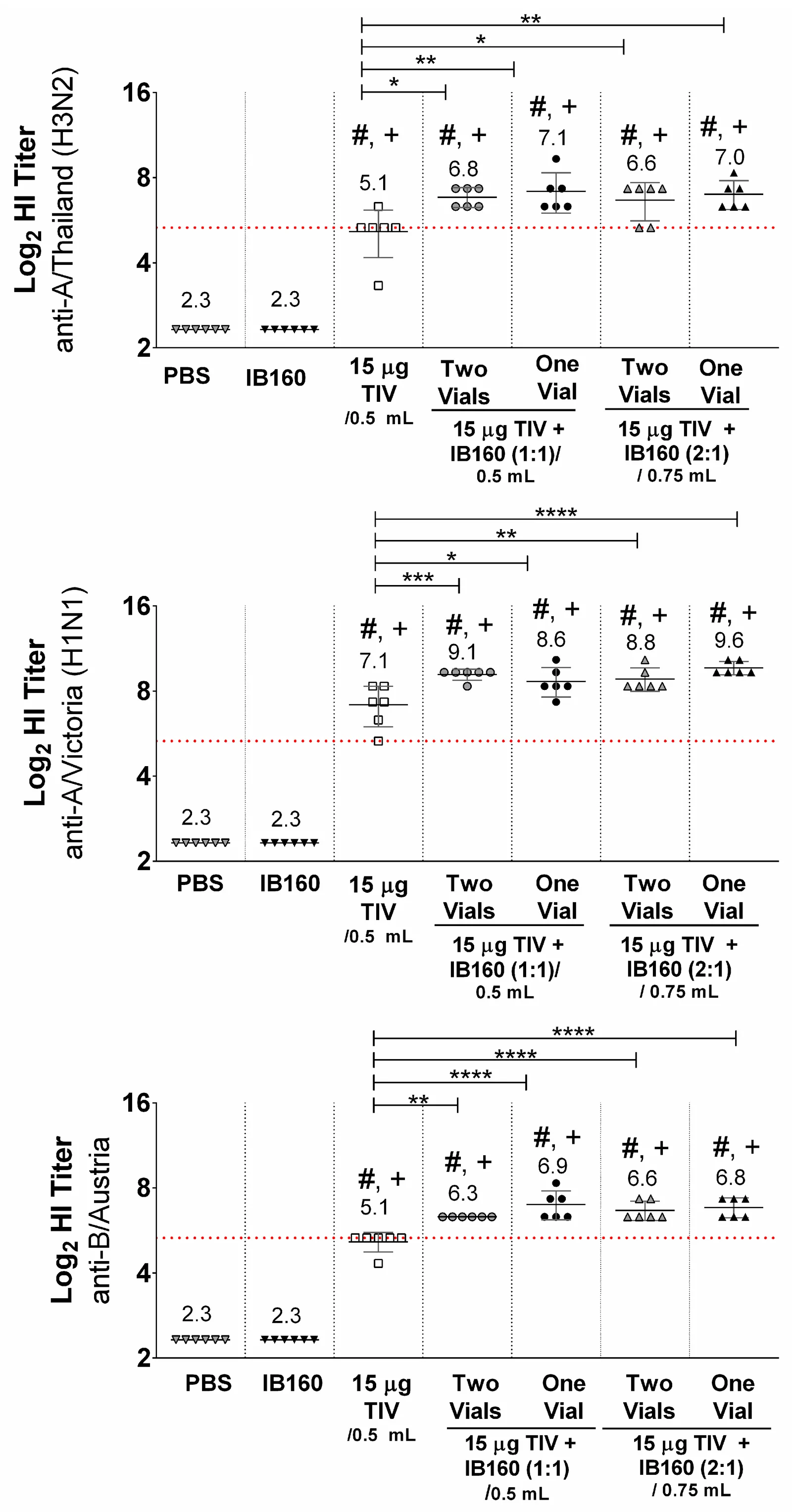
Comparison of two-vial and one-vial formulations of TIV (15 µg, 1:1 and 2:1 ratios) evaluated by HI assay. Each symbol represents an individual animal (*n* = 6 per group) to evaluate biological variability and functional antibody titers. The horizontal red dotted line represents the HI titer threshold; A value equal to or greater than log_2_ 5.22 (corresponding to a 1:40 dilution) is associated with seroprotection. Statistically significant differences between vaccination groups were determined by one-way ANOVA (* *p* ≤ 0.05, ** *p* ≤ 0.01, *** *p* ≤ 0.001, **** *p* ≤ 0.0001). The symbols # and + indicate statistically significant differences compared to PBS and to IB160 alone, respectively. Numbers above the groups represent GMT values.

Given that IB160 was assayed combined with TIV in a two-vial presentation (1:1, *v*:*v*), where the adjuvant and Influenza antigens are mixed immediately prior to immunization, we also evaluated the feasibility and efficacy of a one-vial formulation (co-formulation with both antigen and adjuvant filled in the same vial). This approach aims to improve production efficiency, reduce manufacturing costs, and minimize potential product loss and possible mistakes at the vaccine application sites. Qualitative screening of total IgG binding titers showed comparable profiles between the one-vial and two-vial presentations across all strains (Figure S3). To confirm functional equivalency, HI assays were performed as the primary immunological metric (Figure 2). HI titers confirmed no significant differences between the one-vial and two-vial formulations across all tested strains. Furthermore, significant differences were strictly confined to comparisons between all tested IB160-adjuvanted formulations and the controls (PBS and IB160) or non-adjuvanted 15 µg TIV (Figure 2). Therefore, these results indicate that both formulation strategies of adjuvanted TIV are possible and demonstrate equivalent immunogenicity, whether filling the antigen and adjuvant in the same vial or in a separated 2-vial format.

### 3.3. Formulation Strategies (1-Vial and 2-Vial) Are Stable up to 12 Months

In order to evaluate whether both formulation strategies remain stable in terms of immunogenic response and physicochemical parameters, each formulation was stored at 6 ± 2 °C for 12 months to cover its use during a seasonal TIV immunization campaign. Immunological stability was assessed using functional HI antibody titers as the primary metric, complemented by an initial qualitative screening of strain-specific total IgG levels by ELISA. For physicochemical parameters, we evaluated the specific parameters for either IB160 or the influenza monovalent antigenic contents. As shown in Figure S4, in all timeframes, the immunized animals showed no body weight loss, presenting consistent weight gain throughout the evaluation period, as evaluated by the percentage of animal weight gain. During the assay period, no adverse events were observed.

**Figure 3:**
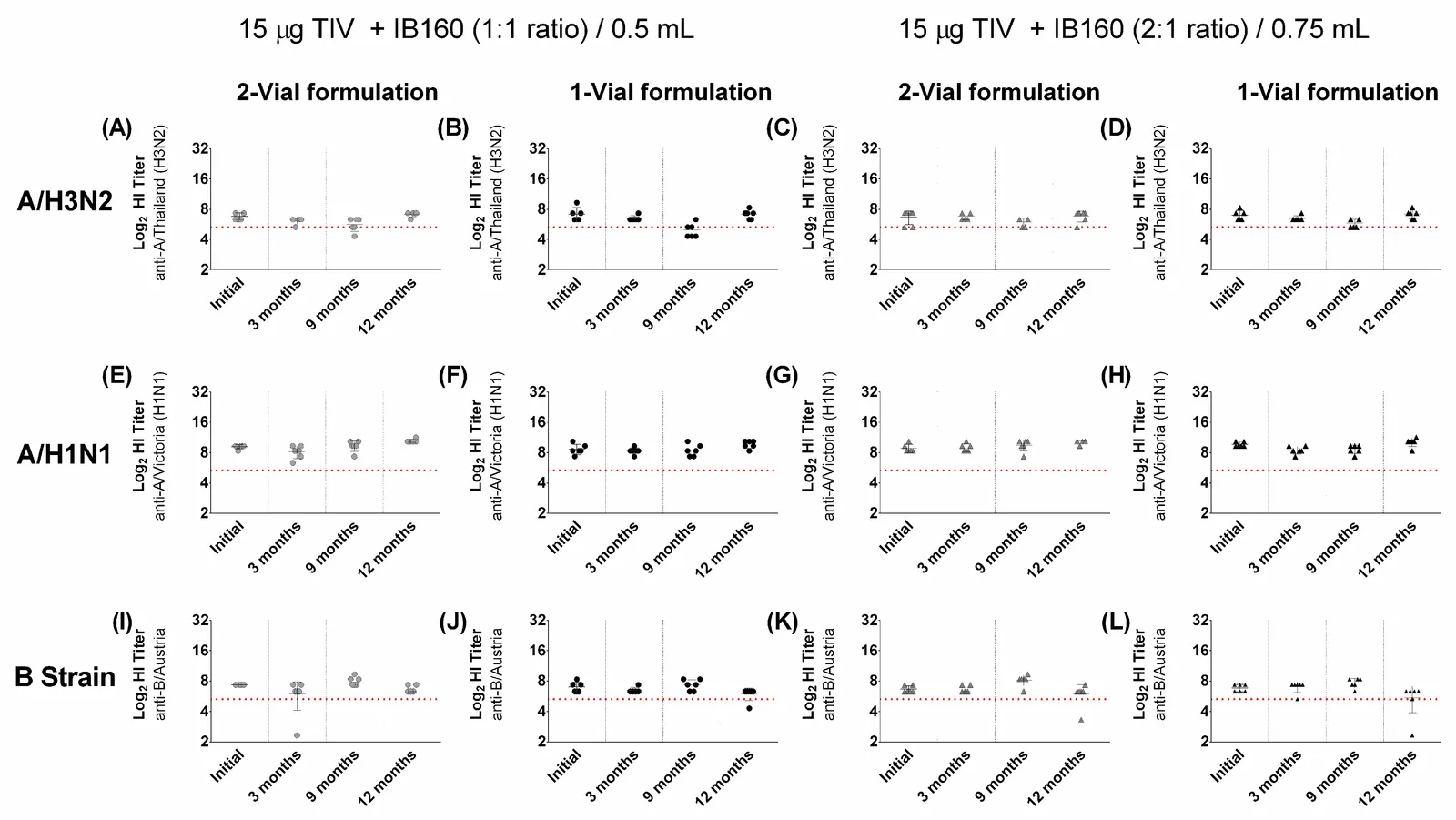
Long-term stability comparison of one-vial and two-vial TIV formulations (1:1 and 2:1 ratios) on log_2_ HI titers. Analysis conducted at baseline (initial), 3, 9, and 12 months showed that all formulations remained stable over 12 months, maintaining comparable functional immune responses observed between one-vial (**B**,**D**,**F**,**H**,**J**,**L**) and two-vial (**A**,**C**,**E**,**G**,**I**,**K**) presentations. Each dot represents an individual immunized animal (*n* = 6 per group), and error bars represent mean ± SD. The horizontal red dotted line represents the HI titer threshold; A value equal to or greater than log2 5.22 (corresponding to 1:40 dilution) is associated with seroprotection.

The evaluation of animal sera after immunization revealed that long-term stability of the formulated vaccines maintained their immunogenicity as presented in 1-vial or 2-vial formulations for up to 12 months regarding total IgG related to each strain (Figure S5), as well as hemagglutination-inhibiting (HI) antibodies determined by HI assay (Figure 3). The analysis was conducted at initial (after filling) and after 3, 9, and 12 months, showing consistent titers over time for both 1-vial and 2-vial formulations.

**Figure 4:**
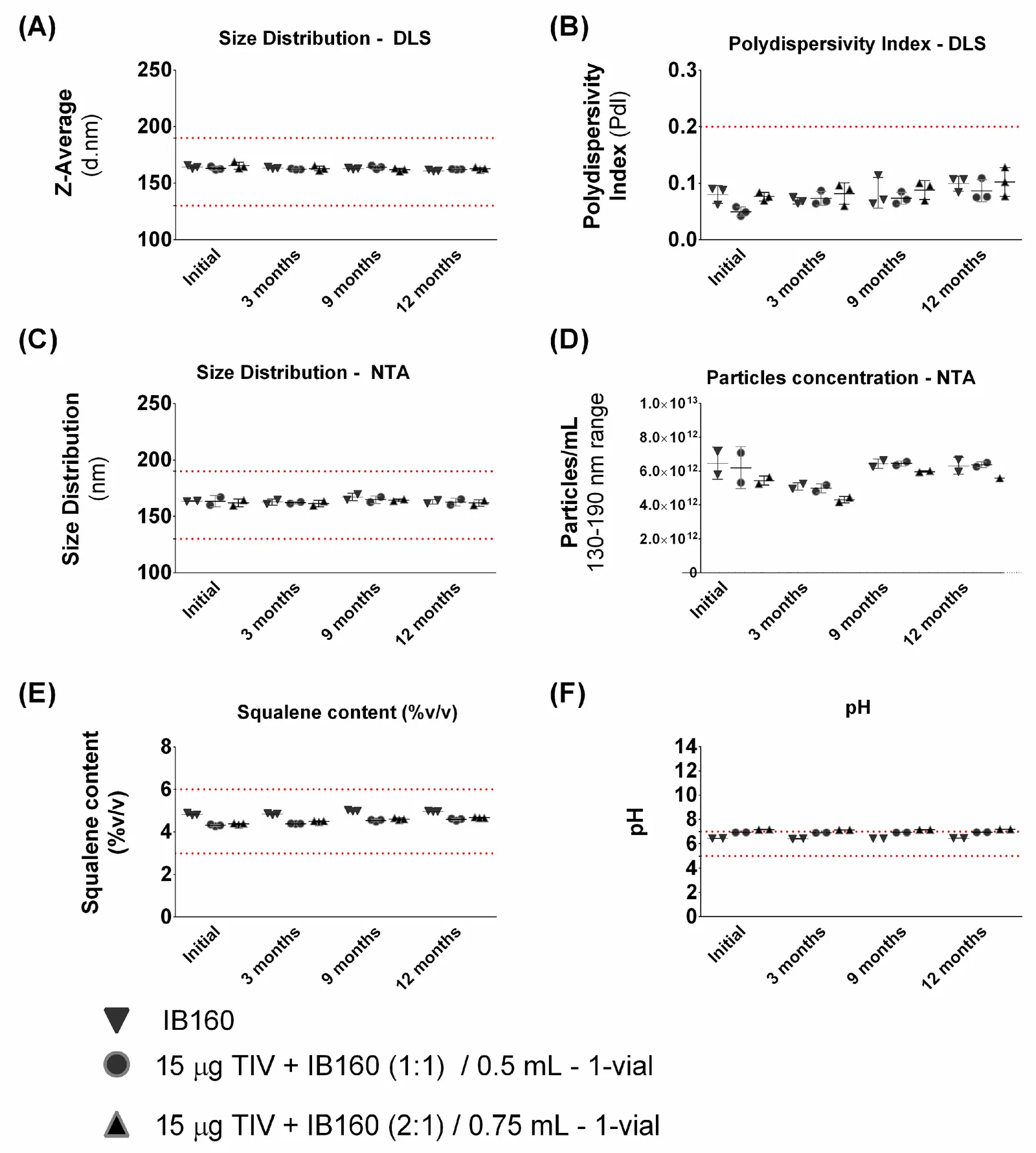
Physicochemical analysis of IB160 and TIV combined with IB160, in a proportion of 1:1 and 2:1 (*v*/*v*), over 12 months. (**A**) Particle size measurement by DLS, performing three reads per sample/stability time, with a specification limit range between 130–190 nm; (**B**) PdI indicates monodispersity of samples throughout the study time. Values below 0.2 indicate monodispersity. USL: upper specification limit of IB160; LSL: lower specification limit of IB160; (**C**) Particle size measurement by NTA, performing three reads per sample/stability time, with a specification limit range between 130–190 nm; (**D**) Particle concentrations obtained by NTA analysis; (**E**) Squalene content determined by HPLC; (**F**) pH determinations. Note: IB160 control was analyzed as the stock emulsion without vehicle dilution, whereas the 1:1 and 2:1 formulation groups contained PBS to adjust the final dosing volume (Table 1). In panels D and E, values for the formulation groups were adjusted by their respective dilution factors (1:2 for 1:1 *v*/*v* ratio and 1:3 for 2:1 *v*/*v* ratio) to reflect equivalent baseline concentrations relative to the bulk IB160 control. Unadjusted raw measured values are reported in Table S1. The red dotted lines represent the upper and the lower specification limit values of IB160. For PdI, monodispersity is below 0.2, as indicated by the red dotted line in panel (**B**).

**Figure 5:**
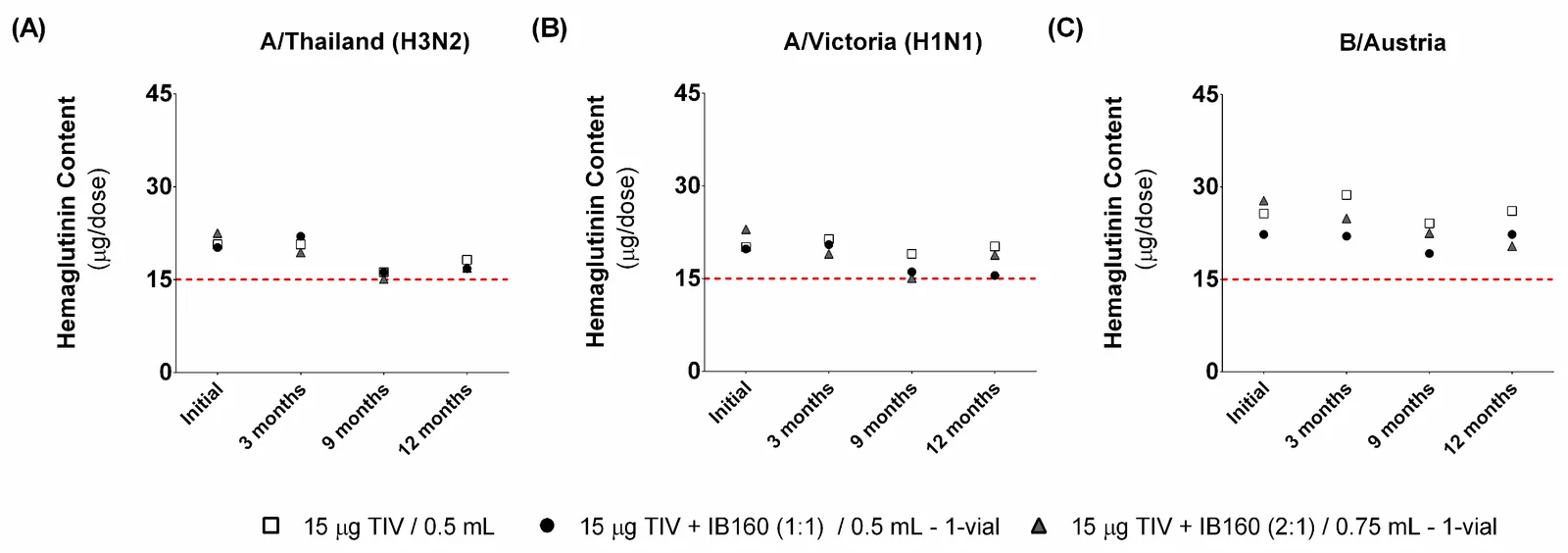
Influenza monovalent strains’ stability on formulations over 12 months. Hemagglutinin content presented in the formulations over time was determined by SRID: (**A**) H3N2; (**B**) H1N1; and (**C**). B strains. The red dashed line represents the minimal antigen content required in the TIV vaccine.

Figure 4 shows that the physicochemical integrity of the IB160 when combined with TIV in a one-vial formulation was preserved and comparable to IB160 alone in the two-vial formulation for up to 12 months. Particle size and squalene content, which are critical specification parameters for IB160 production, remained within acceptable ranges, with no indication of aggregation (PdI < 0.2), over the 12-month period. Moreover, particle concentration analysis in the 130–190 nm range confirmed that the adjuvant remained stable over 12 months in the one-vial IB160 formulations, with similar results as the IB160 vial from the two-vial presentation. Although minor fluctuations occurred, the one-vial formulation showed comparable particle concentrations over the 12-month period as the IB160 vial from 2-vial formulation. To allow direct comparison across mixing ratios against the undiluted IB160 bulk control, data were normalized for dilution factors; actual raw measured values and corresponding normalized concentrations are provided in Table S1. As expected, the 1-vial formulation had the pH of the sample increased to levels closer to the antigens’ pH than to the IB160 emulsion’s pH filled alone in the vial, as previously observed [10], though it did not alter the adjuvant’s physicochemical or immunogenic properties. Lastly, the hemagglutinin content of each Influenza strain was monitored by SRID (Figure 5) and confirmed that the individual monovalent influenza strains inside the ready-to-use 1-vial suffered no antigenic content changes up to 12 months, since the minimal dose was achieved (15 µg) for each monovalent, as observed from the TIV vial from the 2-vial formulation. Altogether, these results show once again that the 1-vial formulation is viable in both 1:1 and 2:1 volume ratios (antigen:IB160, in 0.5 and 0.75 mL final volumes, respectively, as indicated in Table 1), like the 2-vial formulations.

## 4. Discussion

Annual Influenza vaccination remains the main pillar for preventing severe flu symptoms. However, due to poor responder groups, such as the elderly, the inclusion of adjuvants becomes an important strategy to enhance the immunogenicity of Flu vaccines [4,19,20,21]. Adjuvant IB160 has previously demonstrated its ability to enhance the immune response of pandemic influenza candidate vaccines when administered using a 2-vial strategy, mixing the antigens from one vial into the other vial containing the adjuvant emulsion right before immunization [10,15]. However, the switch of strategies from pandemic to routine seasonal vaccination brings new challenges: while pandemic scenarios prioritize rapid deployment and flexible antigen-sparing using two separate vials, as the urgency of the situation makes the time required for long-term co-formulation stability studies more challenging, annual seasonal campaigns demand optimized production and simplified logistics. To address these potential operational and immunological issues, the present study systematically explored formulation possibilities for a seasonal influenza vaccine adjuvanted with IB160 emulsion. We investigated antigen:adjuvant ratios (1:1, 2:1, and 3:1, see Table 1) as well as the standard and higher antigen doses (15 µg and 30 µg, respectively), while evaluating the viability and immunogenicity of a one-vial presentation compared to the two-vial presentation. In addition, we have also evaluated their formulation stabilities for up to 12 months.

Our results revealed no statistical difference between the 15 µg and 30 µg non-adjuvanted formulations in HI titers. This corroborates previous clinical trials which demonstrated that doubling the standard antigen content of Influenza vaccines (from 15 µg to 30 µg) does not improve serum antibody responses, in contrast to that observed for the high-dose formulation approved for use (60 µg/dose, Fluzone, Sanofi), which significantly enhances immunogenicity in the elderly [22,23,24]. However, here we showed that combining the TIV with adjuvant IB160 in a two-vial formulation improved the immunogenic responses, and this enhancement was dependent on antigen:adjuvant ratios. As shown in Figure 2, formulations with a 3:1 ratio (*v*/*v*, 0.5 mL final volume), which had the lowest adjuvant volume (0.125 mL per dose), were not statistically different from non-adjuvanted TIV. On the other hand, formulations of 1:1 ratio (0.5 mL/dose) and 2:1 (0.75 mL/dose), which maintained a constant 0.25 mL volume of adjuvant per dose, showed statistical superiority to non-adjuvanted Influenza vaccine. This performance is similar to the adjuvant dose-response dynamics reported by Hatz et al. (2012) and Della Cioppa et al. (2012), where reduced adjuvant amount was insufficient to reach protective titers, while a maximum adjuvant dose in the formulation tested (1:1) was critical to obtain robust seroprotection [22,25]. Taken together, these findings demonstrate that adjuvant-mediated immune enhancement requires a minimal volume of Squalene-based emulsion adjuvant used in the immunization, probably representing the volume needed to mobilize the immune cells to create a proper immunocompetent environment to elicit the necessary immune responses. When comparing the one-vial (ready-to-use) and two-vial (mixed before immunization) adjuvanted vaccine formulations, no statistically significant differences in the HI-elicited immunogenicity were observed, regardless of the antigen-to-adjuvant ratios tested (1:1 or 2:1). Probably because, in both cases, the volumes used (0.25 mL) of IB160 were the same and enough to mobilize and induce the local immunocompetent environment. Both strategies proved viable and demonstrated clear superiority over the non-adjuvanted control group. Notably, all formulations remained stable for up to 12 months, maintaining HA antigen content, IB160 physicochemical characteristics, and consistent functional HI antibody responses in both one-vial and two-vial presentations (Figure 3), supported by qualitative IgG screening (Figure S5). Similar results were described in a study using recombinant HSV gB2/gD2 vaccine adjuvanted with MF59 emulsion that compared a one-vial formulation against a two-vial presentation (also pre-mixed before immunization), which demonstrated that both formats preserved antigen stability and emulsion characteristics at 4 °C and 25 °C for 90 days. Moreover, both delivery methods yielded identical virus-neutralizing responses and ELISA titers in animal models, confirming that storing the adjuvant together or separately from the antigen does not impact vaccine efficacy; as a result, the authors selected the one-vial format for clinical development [26].

In fact, the one-vial formulation represents the most desirable format from an end-user perspective, since it drastically reduces the risk of mixing errors and simplifies vaccine administration. Furthermore, within the manufacturing sector, the use of a one-vial presentation also reduces operational costs by limiting the physical space and infrastructure required for multiple industrial filling lines and storage spaces [27]. Interestingly, the Fluad vaccine (CSL-Seqirus) is an influenza vaccine indicated for the elderly that combines influenza antigens and the Squalene-based MF59 adjuvant in a pre-filled syringe [28]. Indeed, our results showed that a one-vial formulation is a viable strategy for a pre-mixed TIV adjuvanted with IB160 (an MF59-like adjuvant), which can benefit Flu campaigns, especially targeting the elderly population that requires a stronger immune boost. Nevertheless, the optimization of the one-vial presentation does not undermine the value of the two-vial approach. Instead, it expands the vaccine presentation possibilities: the two-vial strategy remains highly advantageous for pandemic emergencies due to the possibility to stockpile a strategic volume of already filled IB160 for deployment agility and antigen-sparing flexibility without the prerequisite of a long-term stability study, whereas the one-vial presentation stands out as the optimal approach to meet the predictable, high-throughput logistical demands of annual routine seasonal campaigns.

Noteworthy, potential limitations of this study warrant consideration. First, the immunogenicity evaluation relied on a sample size of six rats per group. Although this sample size proved statistically sufficient to detect significant differences owing to low intra-group variance, a larger number of animals could further validate these findings. Second, administering larger injection volumes (0.75 mL for 2:1 ratios) required dividing the dose across both hind limbs (0.375 mL per quadriceps muscle). Although this bilateral intramuscular procedure was well tolerated without visible local tissue damage or weight loss, rats that received a larger vaccine volume (0.75 mL vs. 0.5 mL) may have the immune response affected due to a different extent of local tissue damage.

## 5. Conclusions

The findings reported here show that the IB160 adjuvant-mediated enhancement of immune response in adjuvanted TIV is dependent on the amount of adjuvant used in formulation. The results reinforce that the inclusion of the adjuvant at its effective dose is responsible for a superior immune response of adjuvanted TIV, independent of whether it is formulated in separate vials or in a ready-to-use formulation using one vial. The comparable immunogenic (as determined by HI assays) and stability profiles observed between these formulation strategies highlight the clinical viability and flexibility to use adjuvants in influenza vaccine formulations. Thus, the IB160 adjuvant can be used by different strategies: a highly agile, emergency use in two-vial vaccine presentation for rapid pandemic response and deployment, and also in a one-vial format ready-to-use for optimized seasonal vaccination campaigns.

## Figures and Tables

**Table 1:** Formulations used in the study.

Formulation	Strategy	Dose Volume	Final Composition per Dose		
			TIV (mL)	IB160 (mL)	PBS pH 7.2 (mL)
PBS	1-vial	0.5	-	-	0.5
IB160	1-vial	0.5	-	0.25	0.25
15 µg TIV	1-vial	0.5	0.249	-	0.251
15 µg TIV + IB160 (1:1)	1-vial	0.5	0.249	0.25	0.001
15 µg TIV + IB160 (1:1)	2-vial	0.5	0.249	0.25	0.001
15 µg TIV + IB160 (3:1)	1-vial	0.5	0.374	0.125	0.001
15 µg TIV + IB160 (3:1)	2-vial	0.5	0.374	0.125	0.001
15 µg TIV + IB160 (2:1)	1-vial	0.75	0.498	0.25	0.002
15 µg TIV + IB160 (2:1)	2-vial	0.75	0.498	0.25	0.002
30 µg TIV	1-vial	0.75	0.498	-	0.252
30 µg TIV + IB160 (2:1)	1-vial	0.75	0.498	0.25	0.002
30 µg TIV + IB160 (2:1)	2-vial	0.75	0.498	0.25	0.002

## Data Availability

The data that support the findings of this study are openly available in the repository “Characterization of seasonal trivalent influenza vaccine formulated with different ratios (*v*/*v*) of squalene containing IB160 adjuvant in one or two-vial presentations”. Data will be made available upon request through contact with the corresponding author, with an appropriate data-sharing agreement.

## Supplementary Materials

The following supporting information can be downloaded at https://www.mdpi.com/article/10.3390/vaccines14090789/s1, Figure S1. Experimental design of the immunogenicity and vaccine stability study. Schematic representation of the immunization schedule in male Wistar rats aged 3 months weighing 300–320 g/animal (*n* = 6 per group, 12 groups per time point), with intramuscular administration route (divided across hind limbs), bleeding timeline, and formulation stability assessment time points (0, 3, 9, and 12 months) as indicated. Created with BioRender.com; Figure S2. Effects of TIV:IB160 ratios (*v*/*v*) in two-vial formulation (ELISA, total IgG). Data were obtained using pooled serum samples (*n* = 6 animals/pool) assayed in technical triplicates to evaluate overall binding antibody profiles. Each symbol represents one technical replicate of the analyzed serum pool (mean ± SD); Figure S3. Strain-specific total IgG antibody responses comparing two-vial and one-vial TIV formulations evaluated by ELISA. Total IgG antibody titers against (A) A/H3N2, (B) A/H1N1, and (C) B/Austria strains were determined using pooled serum samples (*n* = 6 animals/group) assayed in technical triplicates to evaluate overall binding antibody profiles; Figure S4. Body weight curves of immunized rats with TIV + IB160 vaccine formulations. The relative weight (weekly weight/initial weight) is expressed in percentage and was analyzed over the experimental period (A. Initial; B. 3 months; C. 9 months; and D. 12 months), according to immunization protocol. The graphics illustrate mean ± standard deviation (SD) of *n* = 6 animals per group receiving each vaccine formulations (15 µg TIV, 15 µg TIV + IB160 (1:1) one-vial, 15 µg TIV + IB160 (1:1) two-vials) or controls (PBS and IB160); Figure S5. Long-term stability comparison of one-vial and two-vial TIV formulations on log2 strain-specific IgG titers. Analysis conducted at baseline (initial), 3, 9, and 12 months showed that all formulations, both one-vial (B,D,F,H,J,L) and two-vial (A,C,E,G,I,K) presentations, remained stable over 12 months, being able to elicit the similar immune response in terms of log2 strain-specific IgG titers; Table S1. Measured (unadjusted) and normalized values for particle concentration and squalene content of IB160 adjuvant formulations during the 12-month stability study. Values represent raw measured data (actual concentrations in vials) and normalized values adjusted by formulation dilution factors (1:2 for 1:1 *v*/*v* ratio and 1:3 for 2:1 *v*/*v* ratio). The IB160 bulk control was evaluated as the stock adjuvant emulsion without phosphate-buffered saline (PBS) vehi-cle addition, representing the pure concentrated component prior to antigen mixing. Formulated vaccines (1-vial format) contained PBS to achieve the final administration volume after antigen addition, as shown in Table 1. Data are presented for for individual duplicates (R1 and R2) or triplicates (R1, R2, and R3), with the mean of replicate measurements highlighted in yellow.

## Abbreviations

TIV	Trivalent Influenza Vaccine
GMP	Good Manufacturing Practice
SRID	Single Radial Immune Diffusion
HA	hemagglutinin
HI	Hemagglutination Inhibition
ELISA	Enzyme-Linked Immunosorbent Assay
