# Supplementary material for: Characterization of Seasonal Trivalent Influenza Vaccine Formulated with Different Ratios (v/v) of Squalene-Containing IB160 Adjuvant in One- or Two-Vial Presentations
Source: Vaccines (Basel). 2026 Sep 9;14(9):789. doi: 10.3390/vaccines14090789 (PMC13611202; doi:10.3390/vaccines14090789)
Supplement: Supplementary file 1 [file vaccines-14-00789-s001.zip › vaccines-4511280-supplementary.pdf]

vaccines

MDPI

Characterization of Seasonal Trivalent Influenza Vaccine Formulated with Different Ratios (v/v) of Squalene Containing IB160 Adjuvant in One- or Two-Vial Presentations

Supplementary Material

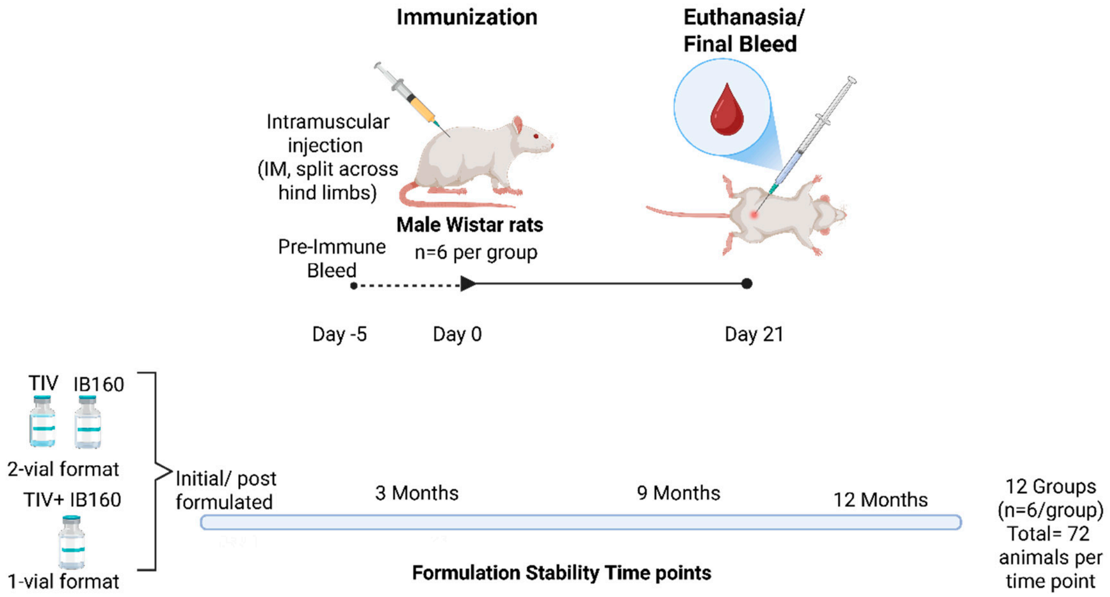

Figure S1. Experimental design of the immunogenicity and vaccine stability study. Schematic representation of the immunization schedule in male Wistar rats aged 3 months weighing 300-320g/animal (*n*=6 per group, 12 groups per time point), with intramuscular administration route (divided across hind limbs), bleeding timeline, and formulation stability assessment time points (0, 3, 9, and 12 months) as indicated. Created with BioRender.com.

Enzyme-Linked Immunosorbent Assay (ELISA)

Total IgG antibodies against each monovalent strain were titrated using ELISA on pooled serum samples (*n* = 6 animals/group, evaluated in technical triplicates) as a preliminary high-throughput binding screening tool. The 96-well plates (Nunc MaxiSorp™ cat#439454, ThermoFisher Scientific, USA) were incubated with 1 µg antigens diluted in PBS, pH 7.4 at 4 °C for 18 h. In this study, plate coating was performed using monovalent bulk antigens corresponding to each of the three constituent strains of the TIV: A/Thailand/8/2022, IVR-237 (H3N2) (batch 2400172/00), A/Victoria/4897/2022, IVR-238 (H1N1) (batch 2301920/00), and B/Austria/1359417/2021, BVR-26, (batch 2302646/00). Blocking the unspecific reactive sites was performed with 1% BSA (m/v) in PBS-T (PBS containing 0.05% Tween 20) at 37 °C for 2 h. Plates were washed three times with PBS-T and incubated with serial dilutions of the pooled

sera in 1% PBS-T BSA for 1 h 30 min at 37 °C. After three additional washes with PBS-T, anti-rat IgG (Sigma-Aldrich, St Louis, MO, USA, Cat# A9037, RRID:AB_258429) conjugated to horseradish peroxidase (HRP) was added, and plates were incubated for additional 1 h at 37 °C. Plates were washed three times with PBS-T, and reactions were developed using 100 µL of o-phenylenediamine dihydrochloride at 0.4 mg/mL (Sigma, St Louis, MO, USA) as substrate in citrate phosphate buffer, pH 5.0. Reactions were stopped by adding 50 µL of 2.0 M H2SO4 (Merck, Darmstadt, Germany). Absorbances were measured in a plate spectrophotometer at 492 nm. The titers were defined as the highest dilution with absorbance, at 492 nm, equal to 0.1.

https://doi.org/10.3390/xxxxx

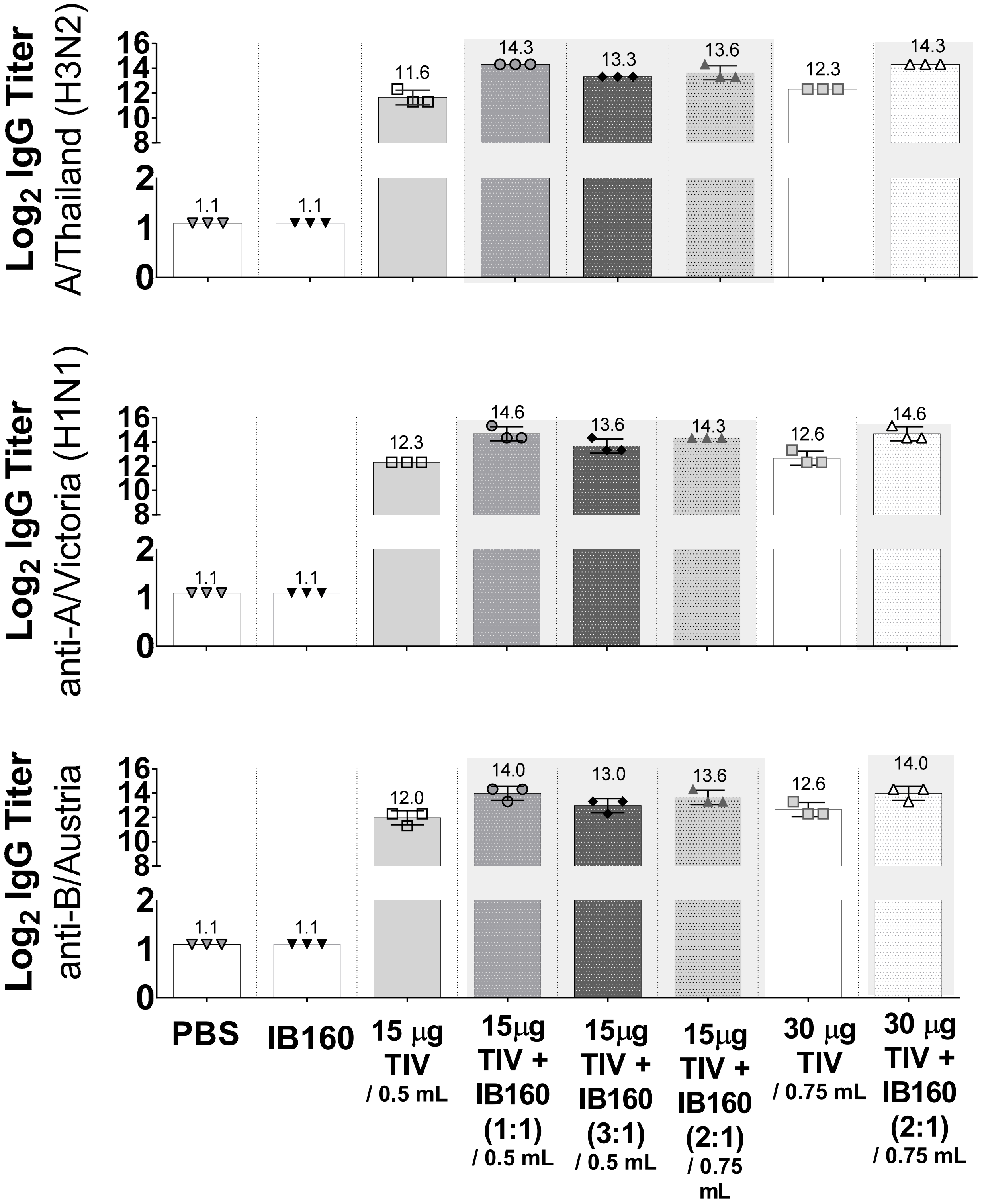

Figure S2. Effects of TIV:IB160 ratios (v/v) in two-vial formulation (ELISA, total IgG). Data were obtained using pooled serum samples (n = 6 animals/pool) assayed in technical triplicates to evaluate overall binding antibody profiles. Each symbol represents one technical replicate of the analyzed serum pool (mean ± SD).

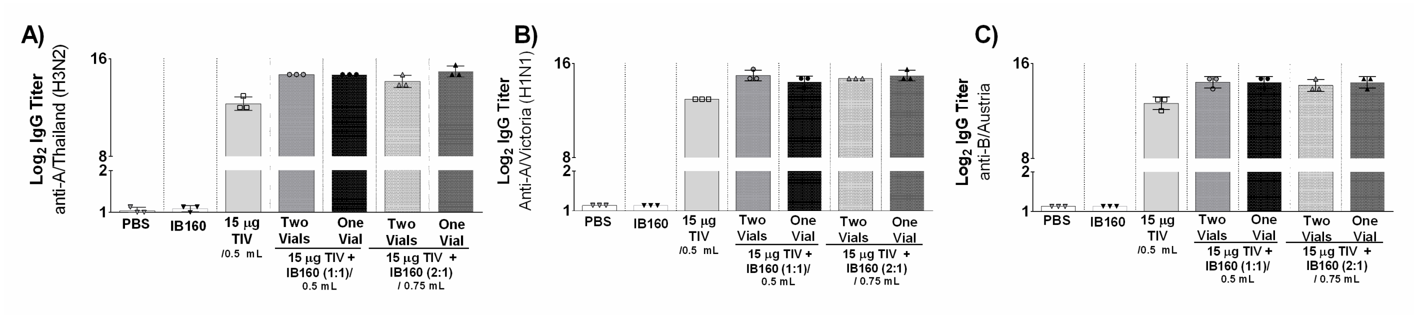

Figure S3. Strain-specific total IgG antibody responses comparing two-vial and one-vial TIV formulations evaluated by ELISA. Total IgG antibody titers against (A) A/H3N2, (B) A/H1N1, and (C) B/Austria strains were determined using pooled serum samples (*n* = 6 animals/group) assayed in technical triplicates to evaluate overall binding antibody profiles

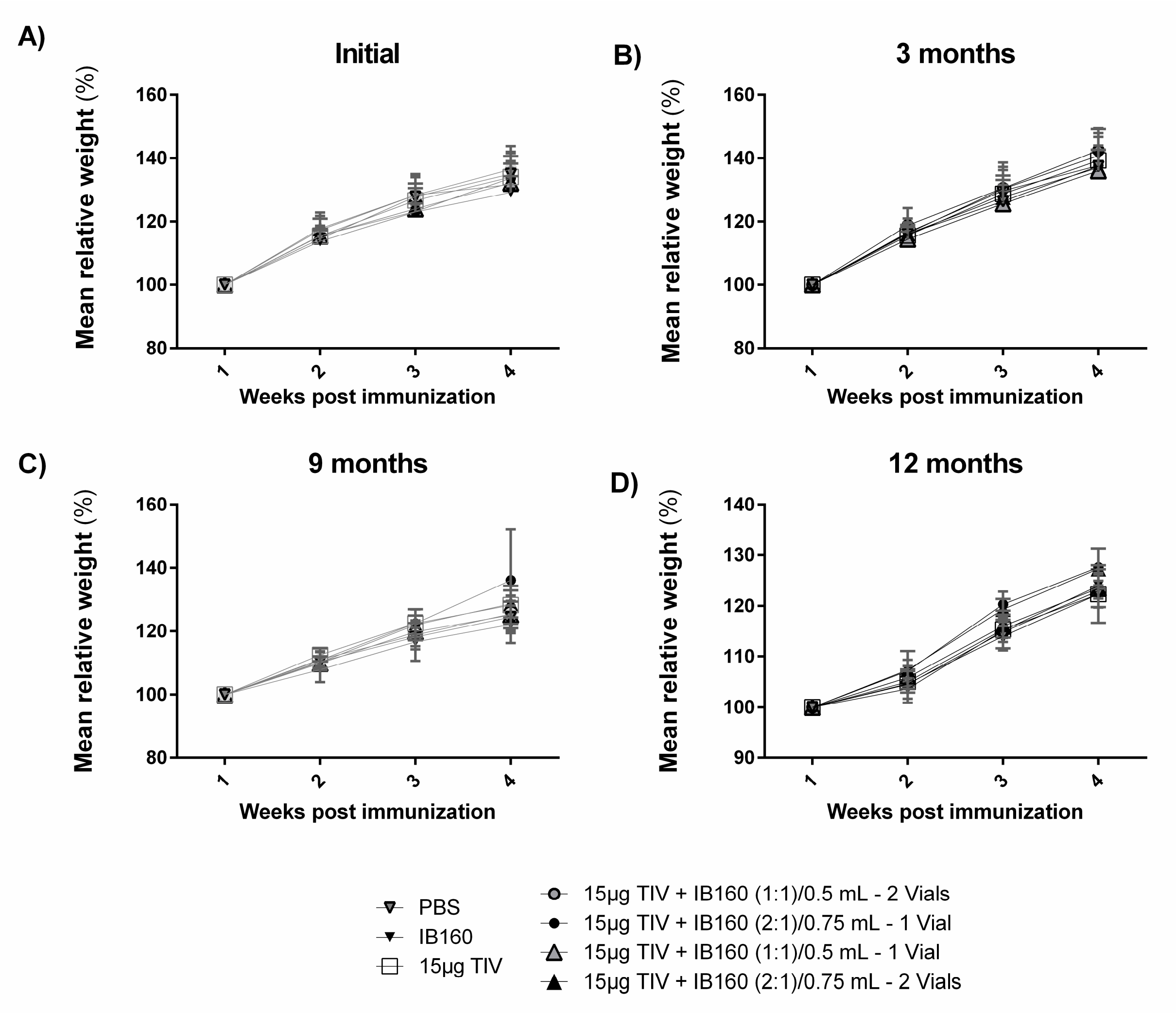

Figure S4. Body weight curves of immunized rats with TIV + IB160 vaccine formulations. The relative weight (weekly weight / initial weight) is expressed in percentage and was analyzed over the experimental period (A. Initial; B. 3 months; C. 9 months; and D. 12 months), according to immunization protocol. The graphics illustrate mean ± standard deviation (SD) of n=6 animals per group receiving each vaccine formulations (15 µg TIV, 15 µg TIV + IB160 (1:1) one-vial, 15 µg TIV + IB160 (1:1) two-vials) or controls (PBS and IB160).

https://doi.org/10.3390/xxxxx

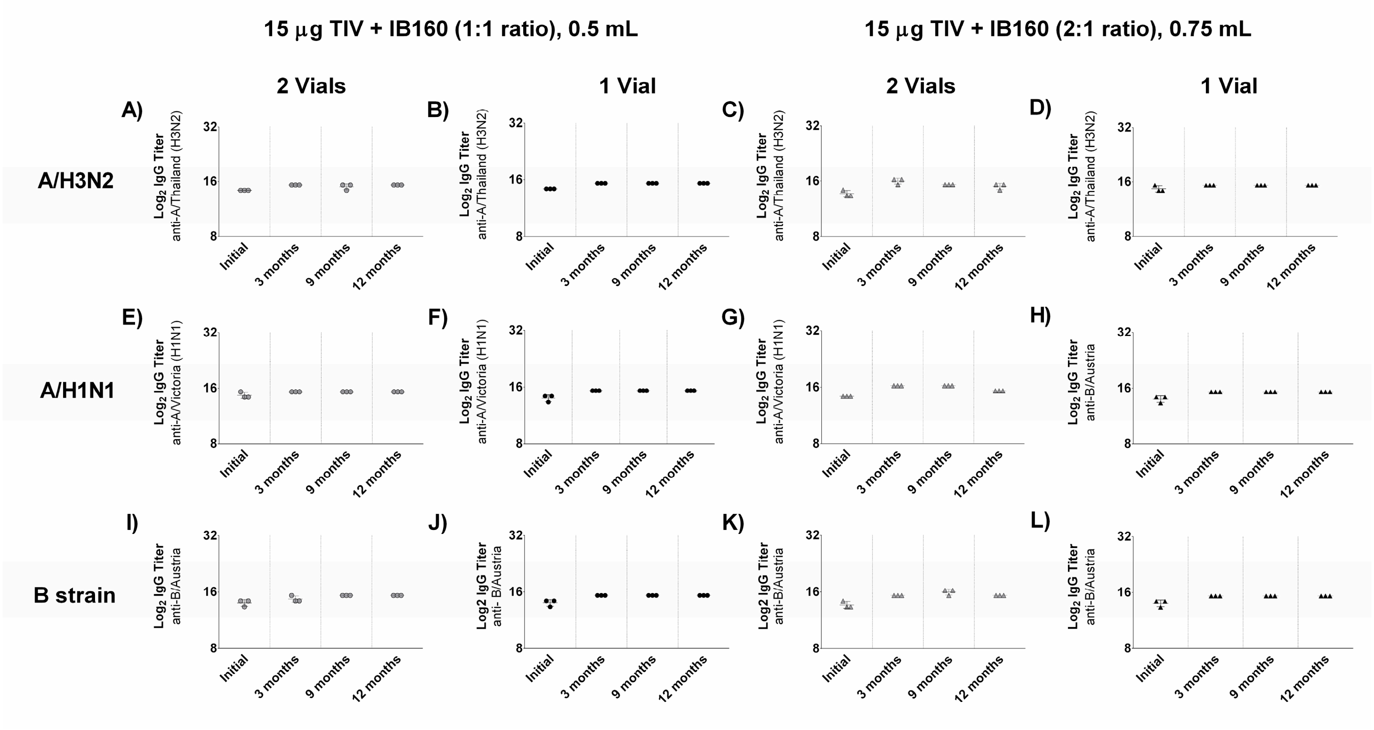

Figure S5. Long-term stability comparison of one-vial and two-vial TIV formulations on log_2 strain-specific IgG titers. Analysis conducted at baseline (initial), 3, 9, and 12 months showed that all formulations, both one-vial (B, D, F, H, J, L) and two-vial (A, C, E, G, I, K) presentations, remained stable over 12 months, being able to elicit the similar immune response in terms of log_2 strain-specific IgG titers.

Table S1. Measured (unadjusted) and normalized values for particle concentration and squalene content of IB160 adjuvant formulations during the 12-month stability study. Values represent raw measured data (actual concentrations in vials) and normalized values adjusted by formulation dilution factors (1:2 for 1:1 v/v ratio and 1:3 for 2:1 v/v ratio). The IB160 bulk control was evaluated as the stock adjuvant emulsion without phosphate-buffered saline (PBS) vehicle addition, representing the pure concentrated component prior to antigen mixing. Formulated vaccines (1-vial format) contained PBS to achieve the final administration volume after antigen addition, as shown in Table 1. Data are presented for for individual duplicates (R1 and R2) or triplicates (R1, R2, and R3), with the mean of replicate measurements highlighted in yellow.

			**Squalene Content (%v/v)**		**Particle concentration (particles/mL)**	
**Storage Time**	**Formulation Group**	**Dilution Factor**	**Measured Value**	**Normalized Value**	**Measured Value**	**Normalized Value**
**Initial**	IB160 bulk (control)	Undiluted	R1: 4.89	R1: 4.89	R1: 5.79E+12	R1: 5.79E+12
			R2: 4.78	R2: 4.78	R2: 7.14E+12	R2: 7.14E+12
			R3: 4.80	R3: 4.80	Mean: 6.47E+12	Mean: 6.47E+12
			Mean: 4.82	Mean: 4.82		
	15 �g TIV + IB160 (1:1)	1:2	R1: 2.13	R1: 4.26	R1: 3.54E+12	R1: 7.08E+12
			R2: 2.15	R2: 4.30	R2: 2.66E+12	R2: 5.32E+12
			R3: 2.17	R3: 4.35	Mean: 3.10E+12	Mean: 6.20E+12
			Mean: 2.15	Mean: 4.30		
	15 �g TIV + IB160 (2:1)	1:3	R1: 1.45	R1: 4.34	R1: 1.75E+12	R1: 5.25E+12
			R2: 1.47	R2: 4.40	R2: 1.88E+12	R2: 5.64E+12
			R3: 1.46	R3: 4.38	Mean: 1.82E+12	Mean: 5.46E+12
			Mean: 1.46	Mean: 4.37		
**3 Months**	IB160 bulk (control)	Undiluted	R1: 4.87	R1: 4.87	R1: 5.25E+12	R1: 5.25E+12
			R2: 4.84	R2: 4.84	R2: 4.95E+12	R2: 4.95E+12
			R3: 4.79	R3: 4.79	Mean: 5.10E+12	Mean: 5.10E+12
			Mean: 4.83	Mean: 4.83		
	15 �g TIV + IB160 (1:1)	1:2	R1: 2.19	R1: 4.37	R1: 2.40E+12	R1: 4.80E+12
			R2: 2.19	R2: 4.39	R2: 2.59E+12	R2: 5.18E+12

			Squalene Content (%v/v)		Particle concentration (particles/mL)	
**Storage Time**	**Formulation Group**	**Dilution Factor**	**Measured Value**	**Normalized Value**	**Measured Value**	**Normalized Value**
			R3: 2.19	R3: 4.38	Mean: 2.50E+12	Mean: 4.99E+12
			Mean: 2.19	Mean: 4.38		
	15 μg TIV + IB160 (2:1)	1:3	R1: 1.49	R1: 4.47	R1: 1.39E+12	R1: 4.17E+12
			R2: 1.50	R2: 4.51	R2: 1.48E+12	R2: 4.44E+12
			R3: 1.50	R3: 4.50	Mean: 1.44E+12	Mean: 4.31E+12
			Mean: 1.50	Mean: 4.49		
**9 Months**	IB160 bulk (control)	Undiluted	R1: 4.98	R1: 4.98	R1: 6.64E+12	R1: 6.64E+12
			R2: 4.96	R2: 4.96	R2: 6.25E+12	R2: 6.25E+12
			R3: 5.02	R3: 5.02	Mean: 6.45E+12	Mean: 6.45E+12
			Mean: 4.99	Mean: 4.99		
	15 μg TIV + IB160 (1:1)	1:2	R1: 2.24	R1: 4.48	R1: 3.17E+12	R1: 6.34E+12
			R2: 2.28	R2: 4.56	R2: 3.28E+12	R2: 6.56E+12
			R3: 2.28	R3: 4.56	Mean: 3.23E+12	Mean: 6.45E+12
			Mean: 2.27	Mean: 4.54		
	15 μg TIV + IB160 (2:1)	1:3	R1: 1.55	R1: 4.65	R1: 1.98E+12	R1: 5.94E+12
			R2: 1.52	R2: 4.56	R2: 2.00E+12	R2: 6.00E+12
			R3: 1.53	R3: 4.59	Mean: 1.99E+12	Mean: 5.97E+12
			Mean: 1.53	Mean: 4.60		
**12 Months**	IB160 bulk (control)	Undiluted	R1: 4.94	R1: 4.94	R1: 5.96E+12	R1: 5.96E+12
			R2: 4.98	R2: 4.98	R2: 6.67E+12	R2: 6.67E+12
			R3: 4.95	R3: 4.95	Mean: 6.32E+12	Mean: 6.32E+12
			Mean: 4.96	Mean: 4.96		
	15 μg TIV + IB160 (1:1)	1:2	R1: 2.30	R1: 4.60	R1: 3.25E+12	R1: 6.50E+12
			R2: 2.26	R2: 4.52	R2: 3.13E+12	R2: 6.26E+12

https://doi.org/10.3390/xxxxx

			Squalene Content (%v/v)		Particle concentration (particles/mL)	
**Storage Time**	**Formulation Group**	**Dilution Factor**	**Measured Value**	**Normalized Value**	**Measured Value**	**Normalized Value**
			R3: 2.31	R3: 4.62	Mean: 3.19E+12	Mean: 6.38E+12
			Mean: 2.29	Mean: 4.58		
	15 μg TIV + IB160 (2:1)	1:3	R1: 1.55	R1: 4.65	R1: 1.86E+12	R1: 5.58E+12
			R2: 1.56	R2: 4.68	R2: 1.86E+12	R2: 5.58E+12
			R3: 1.55	R3: 4.65	Mean: 1.86E+12	Mean: 5.58E+12
			Mean: 1.55	Mean: 4.66		

Note: Measured values reflect the physical concentrations present in the final stored vials. Normalized values reflect the equivalent adjuvant concentration relative to the undiluted IB160 bulk stock, allowing direct comparison across different formulation ratios as displayed in Figure 4.
